# The Amount of Food Ingested and Its Impact on the Level of Uric Acid in the Blood Plasma of Snakes

**DOI:** 10.3390/ani12212959

**Published:** 2022-10-27

**Authors:** Miloš Halán, Lucia Kottferová, Karol Račka, Anthony Lam

**Affiliations:** 1 Department of Epizootiology, Parasitology and Protection of One Health, University of Veterinary Medicine and Pharmacy in Košice, Komenského 68/73, 041 81 Košice, Slovakia; 2 Clinic of Birds, Exotic and Free Living Animals, University of Veterinary Medicine and Pharmacy, Komenského 68/73, 041 81 Košice, Slovakia; 3 Companion Care Vets-Folkestone, Folkestone CT19 5SY, UK

**Keywords:** snakes, feeding, uric acid

## Abstract

**Simple Summary:**

The assessment of uric acid levels in snakes is an important part of the diagnosis of renal diseases. In mammals, lipemic blood from sampling too soon after an animal feeds can have substantial effects on biochemical values. However, fasting status is not routinely considered when sampling reptile blood. The investigation aims to better understand the feed-induced changes that occur and render the analysis of this parameter a more potent diagnostic tool. A study has shown that feeding snakes lead to substantial elevations in uric acid values, with postprandial concentrations significantly elevated for up to 8 days after feeding. To prevent misdiagnosis and distinguish temporary hyperuricemia from clinically relevant increases, it is recommended that sufficient data on time since the last feeding be collected, as well as repeated samples after weeks of fasting.

**Abstract:**

In mammals, lipemic blood from sampling too soon after an animal feeds can have substantial effects on biochemical values. Plasma biochemical values in reptiles may be affected by species, age, season, and nutritional state. However, fasting status is not routinely considered when sampling reptile blood. Assessing uric acid levels in snakes is an important part of the diagnosis of the renal disease. However, the use of this biochemical indicator is undervalued without knowledge of natural uric acid fluctuations and the lack of differentiation from pathological changes. This study aimed to look at the relationship between snake feeding and uric acid concentrations. The investigation aims to better understand the feed-induced changes that occur and render the analysis of this biochemical parameter a more potent diagnostic tool. The study used ten snakes belonging to seven species, and basal uric acid values were evaluated by blood biochemical analysis before feeding. The snakes were fed in two rounds, with successive blood sampling and monitoring of uric acid changes carried out for each. The snakes were fed approximately 50% more with the second round of feeding to investigate the relationship between food supply and uric acid level. The findings show feeding led to substantial elevations in uric acid values, whereby postprandial concentrations were significantly elevated for up to 8 days after feeding. The findings show the significant changes in uric acid levels that occur after feeding and the similarities between postprandial rises in uric acid and those reported in snakes with renal disease. To minimize misdiagnosis and differentiate transient postprandial hyperuricemia from pathological increases, it is recommended that sufficient anamnestic data on time since the last feeding be collected, as well as repeated samples following weeks of fasting. This knowledge is crucial because the amount of feed in terms of intensity and volume has a significant effect on uric acid levels in the blood of snakes.

## 1. Introduction

As most reptiles, snakes tend to mask signs of disease. It is for this reason that many reptiles are not presented to veterinarians early in the course of a disease. This presents a problem for veterinarians in their antemortem diagnosis and delays the initiation of specific therapeutic planning. Although hematology and plasma biochemistry are useful for monitoring animal health, environmental and procedural factors can have a variable effect on reported values. Biochemical analysis is a useful diagnostic tool and an important part of any veterinary diagnosis. However, there are still large gaps in understanding the physiological changes and variables affecting reptile biochemistry, leading to the devaluation of biochemical analysis as a diagnostic tool. With the exponential increase in the number of reptiles, it is becoming increasingly important that our knowledge of these species grows in parallel with their numbers [1].

Metabolic diseases are among the most common problems seen in captive reptiles. Between 1992 and 1996, McWilliams & Leeson [2] reported that 84.4% of lizard patients had a metabolic illness attributed to poor husbandry and nutritional decisions. This trend may improve with increasing reptile owner awareness and improvements in veterinary medicine, but metabolic disease remains a current issue in reptilian care. Although not a common problem in general veterinary medicine, gout is a common affliction in reptilian patients. 

The renal cortex of reptiles is made up of simple nephrons (cortical nephrons) that have a tubular system lacking the loops of Henle; therefore, reptiles are unable to concentrate their urine. Variable amounts of uric acid, urea, and ammonia (nitrogenous waste) are excreted by the reptilian kidneys, depending on the animal’s environment. Freshwater turtles that spend most of their lives in water excrete equal amounts of ammonia and urea, whereas amphibious reptiles excrete more urea [3]. Alligators excrete ammonia and uric acid, and sea turtles excrete ammonia, urea, and uric acid [4]. Land-dwelling or terrestrial reptiles, such as tortoises, produce more insoluble nitrogenous waste in the form of urate salts and uric acid. This is explained by the fact that these reptiles need to conserve water, and soluble forms of nitrogenous waste require large amounts of water for their excretion. Therefore, terrestrial reptiles produce more insoluble nitrogenous wastes, which are eliminated in a semisolid state [5].

In reptiles, uric acid is filtered by the renal tubules. Uric acid and urate salts are insoluble in water and are excreted by the renal tubules. When the level of circulating urate salts or free uric acid rises above the renal capacity to excrete them, hyperuricemia develops. With persistent hyperuricemia, tophi (crystals) are formed which are deposited by the blood-forming crystals in tissues, organs, and joints throughout the body—otherwise known as gout. In reptiles, tophi may precipitate throughout the body, of which the pericardial sac, kidneys, liver, spleen, lungs, subcutaneous tissues, and joints are the most common sites [6].

Gout is categorized into two types: main and secondary. The overproduction of uric acid in primary gout is caused by an innate metabolic imbalance. Secondary gout is caused by chronic diseases that affect the normal balance of uric acid production and excretion (e.g., chronic renal disease, starvation, hypertension, and the use of nephrotoxic pharmaceuticals, such as gentamycin) [1]. Although numerous factors contribute to the development of gout in reptiles, the most frequent causes leading to hyperuricemia and gout include dehydration, renal damage, and excessive consumption of proteins (purine-rich). The latter is particularly associated with herbivorous reptiles fed diets high in animal protein, as seen commonly in green iguanas on diets of cat food [7].

One study exploring the nephrotoxic effects of gentamicin in snakes found that with high doses, snakes presented extensive tubular necrosis within two weeks, and those exposed to high doses developed visceral gout. Dehydration is another factor that may impair renal function. With normal hydration, the nephron actively excretes three times the amount of urates than it would in the dehydrated state [8].

Certain species of reptiles exhibit elevated uric acid levels during hibernation, thought to be due to the reduced renal tubular blood flow at low temperatures experienced during the hibernation period. Physiological rises in uric acid are seen post-prandial in several carnivorous animals, including reptiles and birds, and are attributed to the metabolism of dietary proteins [9].

Current literature suggests the resampling of blood in healthy snakes exhibiting high uric acid levels in an attempt to rule out underlying physiological elevations in uric acid. This, however, postulates a potential problem in current reptilian biochemistry, as the reference ranges provided may be non-sensitive to potential physiological fluctuations in uric acid concentration. In an attempt to compensate, reptilian clinicians require a full patient anamnesis to identify these factors, which they must consider in the evaluation of the biochemistry panel. One, however, questions the reliability of these current methods and whether or not they may be improved [6,9].

Snakes are predominantly sit-and-wait predators, eating large meals after periods of fasting that can last for months. As a consequence of the feeding behavior and physiology in snakes we would expect more evident elevations in uric acid concentrations after feeding when compared to species that have a more regular feeding regime. Carnivorous reptiles generally have higher uric acid concentrations than herbivorous reptiles, and previous studies have shown that postprandial hyperuricemia can result in a 1.5-fold to 2-fold increase in uric acid [4]. A study run by Maixner et al. [10] on five species of reptiles showed postprandial uric acid concentrations to rise to 3-fold basal levels. Similar increases in serum uric acid concentrations have been observed in reptilian patients with renal disease. This highlights the importance of considering postprandial effects on uric acid in reptiles when evaluating plasma biochemistry [11].

Season, sex, age, size, and nutritional state are all environmental and physiological factors that can affect clinical pathology values in reptiles [10,12,13].

Seasons, hormones, and diet (depending on the time of blood collection) have all been documented to have an impact on reptile biochemistry [14,15]. Understanding differences in serum uric acid concentrations in healthy reptiles is critical for assessing hyperuricemia in gout patients. However, the existing literature’s uric acid reference ranges are huge and do not account for such physiological variations, making such data less sensitive and potentially misleading interpretations [16].

The aim of the present study was to evaluate the changes of post-feeding UA levels in a limited number of individuals in 7 species.

## 2. Materials and Methods

### 2.1. Species Studied

Ten adult snakes were used in this study: Burmese python (*Python bivittatus*) Tree Red-tailed boa (*Boa constrictor*), Carpet python (*Morelia spilota*), two Beauty rat snake (*Orthriophis taeniurus friesi*), Eastern kingsnake (*Lampropeltis getula*), Rainbow boa (*Epicrates cenchria*), Four-lined snake (*Elaphe quatuorlineata*).

### 2.2. Feeding and Handling

Individual terrariums with glass fronts and ventilation slits in the side panels were utilized to house the snakes. Each snake was kept in a temperature-controlled environment at around 25 °C (±5 °C). The snakes each had been acclimated to the terrarium conditions for at least 6 months before the study began. Each snake was fasted for at least 14 days to avoid anomalies from prior meal preprandial sampling. Rodents (*Rattus norvegicus*) were given to the snakes, and the rats’ body weights were recorded after first and second meal (Table 1) and they have free access to water. 

### 2.3. Blood Collection and Processing

Before each snake was fed, blood samples were taken and tested to determine baseline uric acid concentrations. Day 0 was the day when we took blood in the morning to determine the current value and the snakes were fed in the afternoon. From the day 1, we monitored the changes in the uric acid level during 9 days. After each meal, blood uric acid concentrations were monitored daily until they reverted to near-baseline values. 

Blood was collected via the ventral coccygeal vein with a 23-gauge needle, and the samples were collected into EDTA tubes. After centrifuging the samples, a volume of 5 μL of plasma was collected using a micropipette and aliquoted for analysis. For the measurement of uric acid concentrations from the serum were performed with the Cobas c111 analyzer, which provided readings for each sample in μmol/l.

## 3. Results

The results in the Table 2 and Table 3 show the variation in plasma uric acid concentration in the snakes before and after feeding.

**Figure 1:**
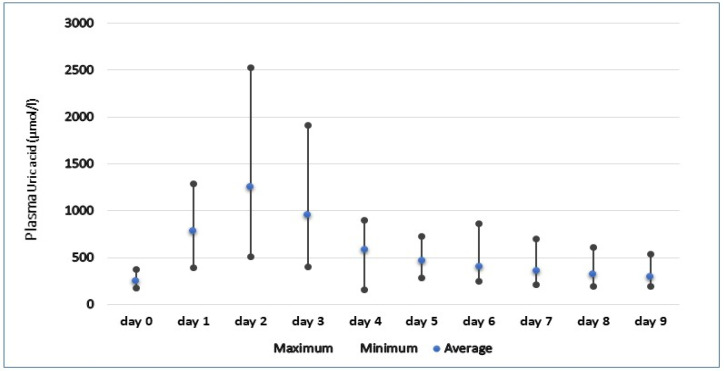
Pre-feeding (day 0) and post-feeding (day 1–9) plasma uric concentration range in snakes after meal 1.

**Figure 2:**
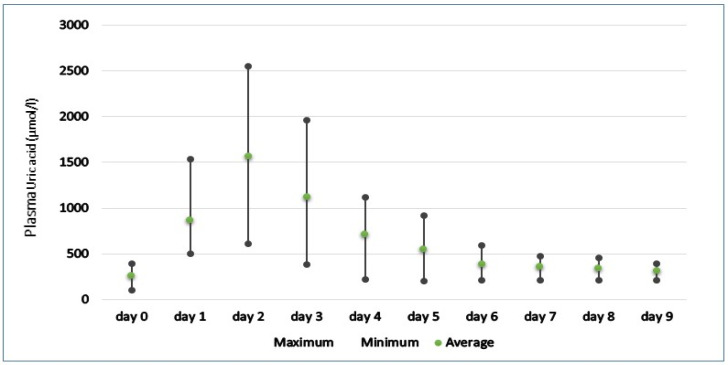
Pre-feeding (day 0) and post-feeding (day 1–9) plasma uric concentration range in snakes after meal 2.

As seen in Figure 1 and Figure 2, the post-prandial levels of uric acid rise substantially in all snakes, with mean peak concentrations reaching 1251.88 and 1568.27 µmol/l in the first and second rounds of feeding, respectively. Plasma uric acid reached peak concentrations between 1 and 2 days in all snakes, with maximum recorded levels reaching 2551.1 µmol/l in *Orthriophis taeniurus friesi* during the second round of feeding. In the snakes studied, the majority showed similar patterns of sharp elevations in uric acid followed by a more gradual decline, returning to basal concentrations approximately nine days after feeding. The only exception to this was seen in the *Python bivittatus* individual 2′ during the first round of feeding, which showed 2 peaks of uric acid before returning to pre-prandial levels. However, during the second round of feeding, this individual followed the same pattern of an initial increase followed by a regression to basal levels, suggesting this snake may have had some internal factor delaying digestion during the first round of feeding.

With the second round of feeding, we find that the pattern of rising and falling of uric acid is similar to that seen in the first meal, but with its differences. However, a return to insignificant concentrations of uric acid did not occur until the eighth day, three more days than in the first feeding, suggesting that snakes fed larger rats took longer to return to basal concentrations. Peak concentrations of uric acid was recorded between 1 and 2 days post-prandial in all snakes, with maximum concentrations reaching 2524.3 µmol/l in the first round of feeding and up to 2551.1 µmol/l in the second. 

## 4. Discussion

In this study, significant post-prandial increases in plasma uric acid content was observed in snakes. The levels of hyperuricemia reported in this study were similar to those found in reptiles with renal disease or gout. Uric acid levels in reptiles with gout are nearly 2-fold greater than baseline levels [3,6], similar to post-prandial concentrations observed in this study. Because of the differences in the physiologies of snake species, Finding an accurate measure of hyperuricemia is challenging. For example, Campbell [3] state that level greater than 892 μmol/l are considered hyperuricemic when compared to Mitchell [17], who gives a level exceeding 600 μmol/l to indicate hyperuricemia. The current study’s post-prandial readings were significantly greater than this level, with maximum concentrations reaching 6 times higher than pre-prandial levels. These findings show that food has a significant impact on uric acid measurements, to the point that the reptile clinician would misinterpret the results as a sign of disease. Although vastly different in their physiology, finding such correlations between diet and levels of nitrogenous by-products in the blood may point out that such relationships have yet to be found in their reptilian counterparts. Maixner et al. [10] conducted a study on black rat snakes to investigate this relationship between meal size and uric acid concentrations and found that the snakes showed greater elevations in plasma uric acid concentrations e.g., when fed one instead of two prey. This has major consequences for veterinarians when it comes to evaluating biochemistry panels in snakes. It emphasizes the significance of obtaining enough anamnestic data before proceeding with further diagnostic procedures. As previously stated, post-prandial effects on serum uric acid concentrations can extend for up to 5 days or more. As a result, it is appropriate to recommend that the anamnesis include information on the time interval since the last feeding. Clinicians should also keep in mind that reptiles with hyperuricemia should be sampled and re-assessed within one week of fasting to provide a more precise picture of uric acid content and avoid misdiagnosis. Our results are representative for snakes, and the same post-prandial elevations in uric acid may not be present in other species of reptiles. Snakes are transient predators that can undergo long periods of fasting, during which basal levels of uric acid are reduced quite significantly. It is for this reason that we may attribute such dramatic changes in uric acid concentrations in these reptiles [18,19]. 

These findings also raise the question of why urate crystals do not form when uric acid levels rise dramatically after feeding. There is no evidence to suggest that urate crystals are deposited in the tissues of snakes under physiological conditions, apparently even under such significant post-prandial elevations. It is for this reason that we may assume that other substances or processes are involved in reducing urate solubility and facilitating deposition. A study carried out by Alvsaker [20] found that there was an absence of albumin and alpha globulin in the plasma of some people suffering from gout. Primary gout in humans, however, is often attributed to hereditary factors, whereas this link has not yet been established in snakes. The most common causes of gout in reptiles are attributed to husbandry or dietary etiologies [21]. 

Similar studies were conducted investigating the effects of protein supply on plasma urea and creatinine concentrations in dogs and minks [19,22]. The results presented a linear relationship between protein supply and plasma urea concentration, and Tauson et al. [23] conclude that consideration of protein intake and time of feeding should be made when interpreting plasma biochemistry. The Parkinson and Mans [24] investigated the effects of cricket ingestion on plasma uric acid concentration in 12 bearded dragons. Results suggested food should be withheld for ≥48 h prior to blood collection if bearded dragons are used to establish reference intervals for plasma uric acid concentration or when obtaining samples for clinical evaluation. 

Although meal size did not significantly affect peak uric acid concentration. This has clinical implications when determining the time interval between re-sampling in hyperuricemic patients, and highlights the potential error if sufficient time is not taken. Based on the present results, it is evident that re-sampling after 8 days would allow for a more accurate interpretation. However, many variables that would affect these results were controlled, including temperature and access to water. Snakes are poikilothermic and are dependent upon their environmental temperature to fuel their metabolism. An investigation into the effect of temperature on metabolic rate in the *Boa constrictor amarali* found that ambient temperature had a profound effect on the duration of digestion and also digestive efficiency [25]. From this, we can assume that under conditions with more variable temperatures, in particular lower temperatures (not uncommon in captive reptile husbandry), we may see changes in the relationship between feeding and uric acid concentration. Unlike in the wild, snakes in a captive environment are feed on a more regular basis, and smaller species of snake are often fed weekly, approximately the same time interval as it took the snakes in this study to return to basal levels of uric acid. Consequently, it seems that without sufficient time intervals between feeding, basal levels of uric acid would not be reached and the individual would be in a persistent state of mild to moderate hyperuricemia. This underlines another difficulty faced by veterinarians when trying to obtain accurate representations of uric acid levels in snakes and that a period of fasting in these patients would be required to allow for more accurate interpretation.

## 5. Conclusions

Our research shows how feeding influences plasma uric acid content in snakes and emphasizes the need to recognize this link when evaluating clinical chemistry in snakes. This information has major consequences for veterinarians when it comes to evaluating biochemistry panels in snakes. It emphasizes the significance of obtaining enough anamnestic data before proceeding with further diagnostic procedures. As previously stated, post-prandial effects on serum uric acid concentrations can extend for up to 5 days or more. As a result, it is appropriate to recommend that the anamnesis include information on the time interval since the last feeding. Clinicians should also keep in mind that reptiles with hyperuricemia should be sampled and re-assessed within one week of fasting to provide a more precise picture of uric acid content and avoid misdiagnosis.

## Figures and Tables

**Table 1:** Weight of rats fed to each snake for both rounds of feeding.

Species	Rat Weight (g)	
	Meal 1	Meal 2
*Python bivittatus* individual 1′	90	130
*Python bivittatus* individual 2′	120	190
*Python bivittatus* individual 3′	200	310
*Morelia spilota*	160	240
*Orthriophis taeniurus friesi* individual 1′	60	80
*Orthriophis taeniurus friesi* individual 2′	80	120
*Elaphe quatuorlineata*	50	80
*Lampropeltis getula*	40	60
*Epicrates cenchria*	40	70

**Table 2:** Pre-feeding (day 0) and post-feeding (day 1–9) uric acid concentrations in snake plasma after meal 1.

	Plasma Uric Acid (µmol/l)									
Day	0	1	2	3	4	5	6	7	8	9
*Python bivittatus* individual 1′	176.2	392.3	578.4	397.3	361.1	329.2	250.1	212.2	197.0	191.4
*Python bivittatus* individual 2′	349.8	535.9	738.5	891.1	784.2	730.6	866.5	697.5	611.9	536.4
*Python bivittatus* individual 3′	233.8	1115.8	1284.8	1030.3	897.9	526.8	370.1	314.0	270.8	253.1
*Boa constrictor*	370.7	653.6	1926.4	1147.5	741.9	408.3	328.5	373.1	348.7	343.5
*Morelia spilota*	259.6	1010.3	2524.3	1912.8	824.0	571.8	425.5	350.1	285.3	267.9
*Orthriophis taeniurus friesi* individual 1′	277.9	1246.4	1617.2	1050.9	602.3	343.8	333.7	299.3	289.2	284.2
*Orthriophis taeniurus friesi* individual 2′	254.3	404.0	1314.1	1116.5	446.2	279.1	264.4	219.3	218.4	223.6
*Elaphe quatuorlineata*	193.6	1286.5	1333.4	1091.2	814.6	579.3	389.4	439.6	389.4	292.5
*Lampropeltis getula*	238.9	622.8	508.5	417.5	221.6					
*Epicrates cenchria*	175.3	578.9	739.5	538.3	152.8					

**Table 3:** Pre-feeding (day 0) and post-feeding (day 1–9) uric acid concentrations in snake plasma after meal 2.

	Plasma Uric Acid (µmol/l)									
Day	0	1	2	3	4	5	6	7	8	9
*Python bivittatus* individual 1′	297.8	682.7	894.7	723.5	463.2	391.4	324.7	351.7	339.1	312.3
*Python bivittatus* individual 2′	396.5	650.6	1011.4	486.3	330.3	357.8	265.3	294.9	271.6	295.3
*Python bivittatus* individual 3′	352.2	886.0	2057.8	1255.4	682.5	435.2	358.6	458.1	412.6	395.2
*Boa constrictor*	370.7	653.6	1926.4	1147.5	741.9	408.3	328.5	373.1	348.7	343.5
*Morelia spilota*	259.6	1010.3	2524.3	1912.8	824.0	571.8	425.5	350.1	285.3	267.9
*Orthriophis taeniurus friesi* individual 1′	212.0	1033.5	2551.1	1957.5	1092.7	688.0	376.9	333.3	350.1	314.6
*Orthriophis taeniurus friesi* individual 2′	216.2	1234.3	1935.3	1951.7	1006.7	702.3	308.2	306.1	271.7	289.2
*Elaphe quatuorlineata*	298.1	1530.7	1388.7	1053.1	819.6	736.8	593.3	471.4	457.7	329.6
*Lampropeltis getula*	99.1	497.0	982.1	976.7	1011.7	921.9	495.9	431.6	400.7	308.3
*Epicrates cenchria*	203.5	549.5	609.2	387.3	216.9	201.6	213.9	210.8	210.7	208.2

## Data Availability

The data presented in this study are available on request from the corresponding author.
